# Revolutionizing elderly care: Building a healthier aging society through innovative long‐term care systems and assessing the long‐term care acceptance model

**DOI:** 10.1111/ggi.14856

**Published:** 2024-04-07

**Authors:** Chaturapron Chokphukhiao, Wonn Shweyi Thet Tun, Sakaowrat Masa, Somporn Chaiayuth, Jugsun Loeiyood, Cholatip Pongskul, Rina Patramanon

**Affiliations:** ^1^ Information Technology International Program, College of Computing Khon Kaen University Khon Kaen Thailand; ^2^ Center of Excellence in Digital Innovation, Faculty of Education Khon Kaen University Khon Kaen Thailand; ^3^ Khon Kaen University Phenom Center Khon Kaen University Khon Kaen Thailand; ^4^ Department of Chemistry, Faculty of Science Khon Kaen University Khon Kaen Thailand; ^5^ Division of Public Health and Environment Service, Office of Public Health and Environment Khon Kaen Municipality Khon Kaen Thailand; ^6^ Division of Information and Communication Technology Khon Kaen Provincial Health Office Khon Kaen Thailand; ^7^ Department of Medicine, Faculty of Medicine Khon Kaen University Khon Kaen Thailand

**Keywords:** elderly people, long‐term care (LTC) system, technology acceptance model

## Abstract

**Aim:**

With a growing elderly population, the demand for caregivers is increasing in Khon Kaen, Thailand, with approximately 17 000 elderly residents. This growing number of older people and a shortage of caregivers could overload the healthcare system.

**Methods:**

The present study involved 129 healthcare volunteers (caregivers for questionnaires study) and the collection of health data from 290 elderly residents from northeastern Thailand. After training, the volunteers assessed its usefulness through questionnaires. Tool reliability and statistical hypotheses were tested using stratified regression analysis (hierarchical regression) and multiple regression.

**Results:**

The relative mean scores of perceived usefulness, perceived ease of use, attitude toward usage and behavioral intention to use technology were 4.51, 4.29, 4.44 and 4.41, respectively. In addition, perceived usefulness and user attitudes positively affected volunteers' willingness to use the system.

**Conclusion:**

The study was developed from the awareness of enhancing community quality and ecosystem through a long‐term care system application. Analyzing external factors can enhance technology's future effectiveness. **Geriatr Gerontol Int 2024; 24: 477–485**.

## Graphical abstract

In this study, a digital innovation model for long‐term care is investigated, emphasizing technology and community integration. Thestudy involved 129 healthcare volunteers and collected health data from 290 elderly residents in northeastern Thailand. After training,the usefulness and technology acceptance model (TAM) is assessed using questionnaires and statistical analysis.

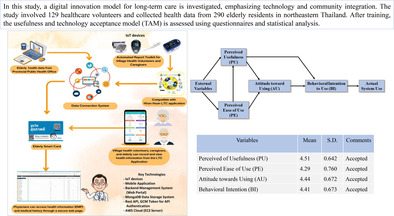

## Introduction

Recently, smart city designs have prioritized aging populations' needs through housing, community interactions, healthcare and social support, integrating information and communication technology for enhanced connectivity and enhanced urban life. City data platforms use advanced analytics, visualization, and machine learning for data‐driven decisions and quality improvement.^1^, ^2^, ^3^, ^4^ Cities are utilizing smart living solutions, such as assistance robotics, wearable tech, remote monitoring and smart home gadgets, to identify health patterns, estimate illness outbreaks and provide individualized healthcare to seniors, including fall sensors and the Internet of Things (IoTs).^5^, ^6^, ^7^ IoT is a network of interconnected devices that communicate and share data over the Internet, enabling remote monitoring, control, and automation of various processes and tasks.^8^

Nowadays, creating digital ecosystems is crucial for sustained competitiveness and fostering innovation. Healthcare's digital transformation, especially through mobile health (mHealth), provides cost‐effective means for preventing and controlling non‐communicable diseases among vulnerable groups, such as the elderly.^9^, ^10^, ^11^, ^12^, ^13^, ^14^ Long‐term care (LTC) means a variety of services that can assist people who have a chronic illness or disability and cannot take care of themselves for a long period of time with both their medical and non‐medical requirements.^15^ LTC focuses on personalized, coordinated services for patient independence, enhances quality of life and promotes the well‐being of older adults.^16^, ^17^, ^18^

Northeastern Thailand's largest region, home to one‐third of the country's population, faces poverty and weak natural resources due to young migration and neglect of older adults. Aging is a key concern for healthcare strategies.^19^, ^20^, ^21^, ^22^, ^23^ The present study establishes a framework for designing a healthy aging society. It specifically addresses the LTC system in northeastern Thailand, where the elderly population is growing and lifespans are increasing.^24^ After introducing LTC, assessing satisfaction through the technology acceptance model (TAM) becomes important. TAM evaluates user adoption, emphasizing perceived usefulness and ease of use.^25^, ^26^ The objectives of the present study are multifaceted: to facilitate the collection of health data, standardize elderly care in northeastern Thailand, contribute to the development of a ‘Healthy Smart City,’ create an automated health analysis system, improve the well‐being of older people, establish sustainable healthcare practices and assess technology acceptance of LTC systems. The TAM is an essential tool in this assessment, which evaluates user adoption by emphasizing perceived usefulness and ease of use.

## Methods

### Operation system of LTC application

**Figure 1:**
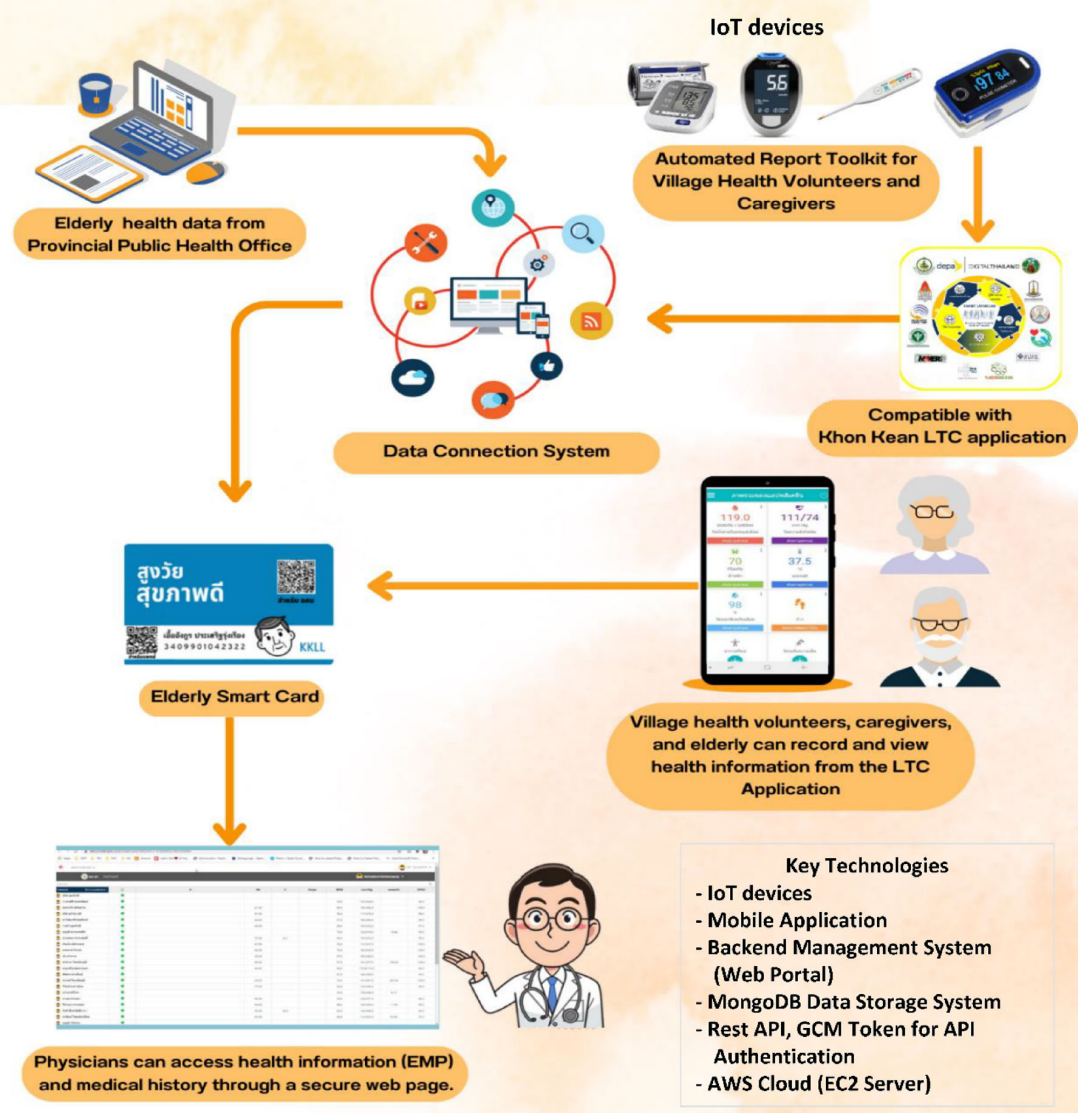
Schematic diagram illustrating the collecting system of health information data from older persons in Khon Kaen Province. Consultation meetings were held with key stakeholders, including Khon Kaen Municipality, Khon Kaen Hospital, the provincial public health office and the Office of Digital Economy Promotion, as shown in Figures 1 and 2. These interactions aimed to enhance the project, which also involved planning and collaboration. IoT, Internet of Things; LTC, long‐term care.

**Figure 2:**
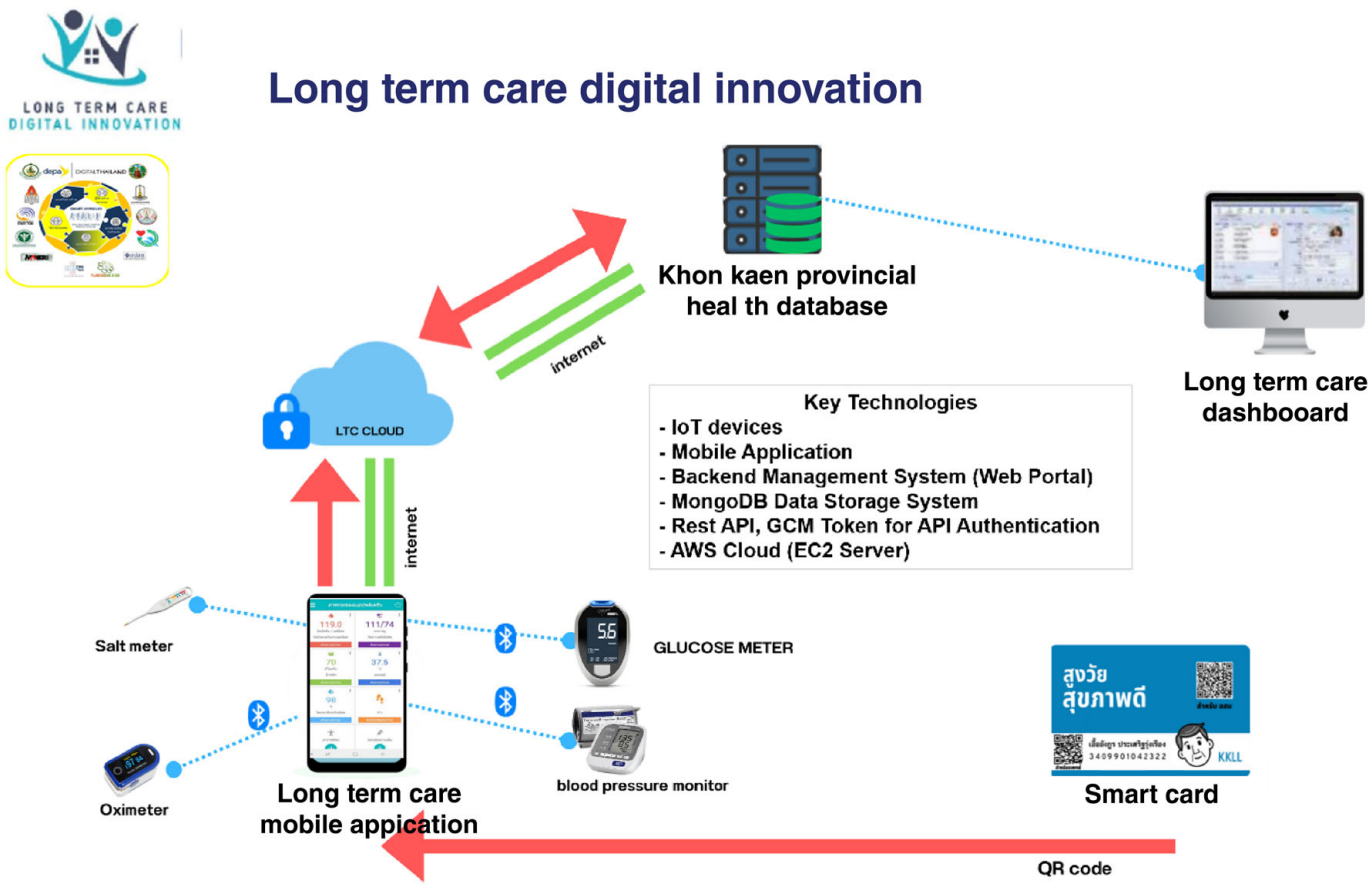
The long‐term care (LTC) digital innovation model system's overall structure, including key technologies and the integrated community ecosystem for a healthy aging society. IoT, Internet of Things.

LTC emphasizes independence, autonomy and dignity for those unable to self‐care, prioritizing quality of life.^27^ The collected health data were recorded to automated input health data (Fig. 1). Central to our approach is IoT devices, including a blood pressure monitor, a glucose meter for blood sugar monitoring, an oximeter for real‐time blood oxygen level monitoring and an electronic salinity meter for quick salinity assessments. The LTC application on mobile devices aids village health volunteers (VHVs) and caregivers by providing a centralized platform for health data storage and instant report generation for elderly patients (Fig. 2). The system's structure consists of four key components: a mobile application, a backend management system (Web Portal), a MongoDB data storage system and a Rest API for seamless data exchange. Data security is upheld through the use of GCM Token for API authentication. The infrastructure is backed by an EC2 server in the AWS cloud, ensuring resource scalability and data accuracy through rigorous testing.

### Recruitment criteria of the healthcare volunteers and collection of health data

Healthcare volunteers were selected based on background, qualifications and experience, prioritizing expertise, and a genuine interest in the well‐being of older adults. Training covered the LTC application, data collection, ethics and patient interaction. Proficiency assessments ensured data quality, with additional support provided as needed. Health data collection of 290 older adult participants took place across five medical centers: Prachasamosorn, Nong Yai, Mittrapad, Wat Nong Waeng and Chata Padung. The volunteers assessed the LTC system satisfaction and acceptance. The older adults were selected based on specific health conditions for LTC technology, excluding those outside the defined age range, residents not in the study area and those without specified health conditions.

### Sample size determination in LTC study

Healthcare volunteers received smart technology and LTC system training, gaining real‐workplace experience. The sample size was determined through G*Power package's analysis, using Cohen's formula^28^ by setting the parameters as follows: the level of significance or *α* equals 0.01 (99% confidence level); power of test or (1–*β*) equals 0.99 (probability of judgment correct); the effect of size or L_2_ is a statistic used to tell the size of the difference when effect of size is equal to 0.15 from the suggestion of using the effect of size when the dependent variable is expected to moderately influence the dependent variable; the numbers of variables used is equal to four variables (Fig. S2). As a result of these calculations, the researchers obtained a sample group consisting of 129 people including caregivers and elderly participants, to assess the reliability of the questionnaire used in the study.

### Data collections of the study

Various health data of 290 elderly adults were measured, collected and recorded through a LTC application, as shown in Fig. 2 and Table 1. Participants were selected based on age, residency and health status, with those outside the defined age range excluded. The study aimed to address chronic illnesses and specific health needs that could benefit from the LTC system. The LTC application linked caregivers to local doctors through QR code‐enabled smart cards to access medical information, aiding diagnosis. Vitally, this study complied with ethical standards, and was authorized by Khon Kaen University (HE622163) and the IRB (IRB00008614). All participants in the present study were informed to provide consent, emphasizing transparency and moral accountability following the International Council for Harmonization of Technical Requirements for Pharmaceuticals for Human Use Guideline for Good Clinical Practice.

**Table 1:** Demographic and disease/disorder classification of older patients for the long‐term care system

	*n*	Percentage (%)
Sex		
Male	14	10.9
Female	115	89.1
Age		
21–30 years	4	3.1
31–40 years	5	3.9
41–50 years	7	5.4
51–60 years	42	32.6
>60 years	71	55
Occupation		
Housewife/butler	57	44.2
Retirements	10	7.8
Employees of privates	1	0.8
Employees of companies	16	12.4
Hired workers	36	27.9
Freelance/trade	7	5.4
Unemployed	2	1.6
Education level		
Lower than a bachelor's degree	109	84.5
Bachelor's degree	17	13.2
PhD	3	2.3
Income		
<15 000 baht	103	79.8
15 000–30 000 baht	17	13.2
30 000–50 000 baht	5	3.9
>50 000 baht	4	3.1
Positions		
VHVs	103	79.8
VHVs and CG	26	20.2
Medical centers where volunteers were affiliated		
Prachasamosorn medical center	29	22.5
Nong Yai medical center	39	30.2
Mittrapad medical center	20	15.5
Wat Nong Weang medical center	19	14.7
Chata Padung medical center	22	17.1
Disease and abnormality		
Hypertension	65	22.41
Diabetes	43	14.82
General physical examination/dental examination/screening	34	11.72
Mental and behavioral disorders	26	8.96
Eyes and vision impairment	13	4.48
Fever/flu/cough/flu vaccination	11	3.79
Chronic ischemic heart disease	10	3.45
Supervise dressings and sutures/relieve symptoms	9	3.1
Muscular dystrophy symptoms	7	2.41
Prostate hypertrophy	7	2.41
Osteoporosis	5	1.72
Lung and respiratory disorders	5	1.72
Malignant tumors	5	1.72
Asthma	4	1.37
Paralysis	3	1.03
Hyperlipidemia	3	1.03
Gout	1	0.34
Parkinson's disease	1	0.34
Others	35	12.06
No information	7	2.41

Abbreviations: CG, caregivers; VHVs, village health volunteers.

### Evaluation of usefulness and validation of questionnaires

**Figure 3:**
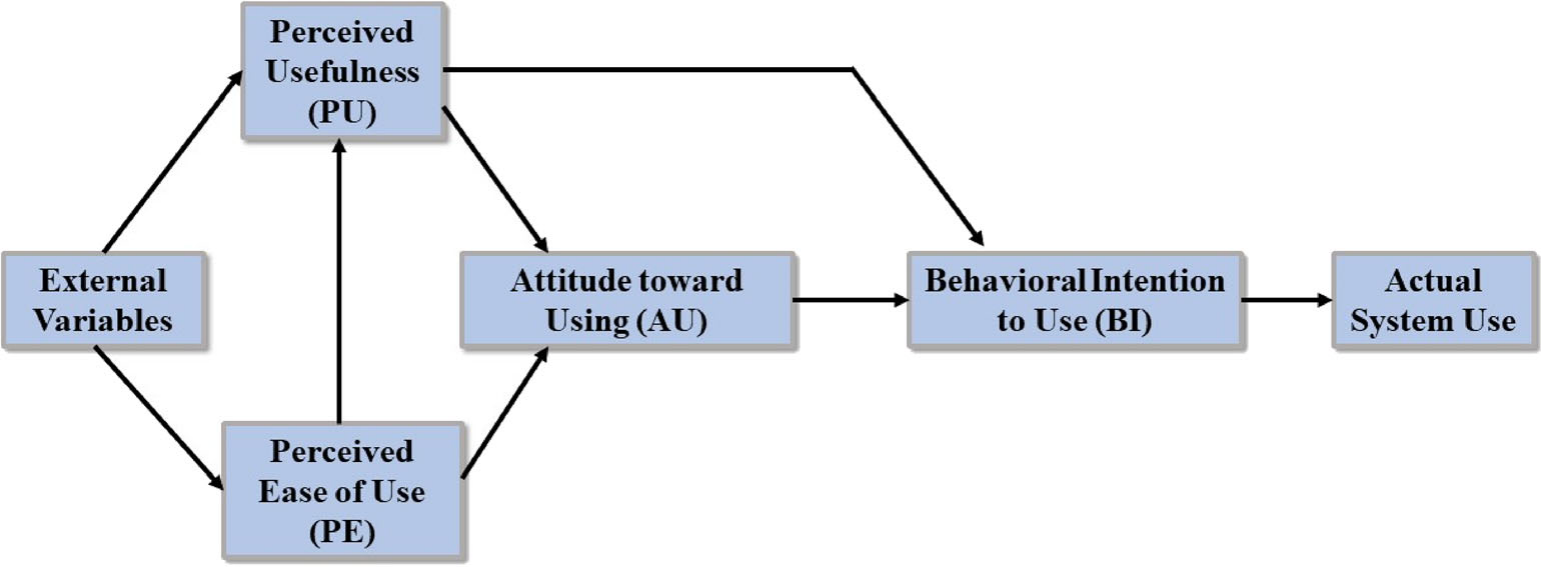
Technology acceptance model used in the study to investigate multiple factors: (1) perceived usefulness of technology, (2) perceived ease of use of technology, (3) attitude to use, and (4) behavioral intention.

To assess VHVs' intention to use the LTC system, we employed modified technology acceptance questionnaires (Table S1) based on the technology acceptance model. The study used a widely used and validated questionnaire from Davis et al.,^25^ which has a history of application and validation in various contexts, especially in healthcare services (Table 2), ensuring its reliability and validity in measuring perceived usefulness and ease of use. In addition, the project director, committee boards with medical professionals and researchers participated in validating and confirming the suitability of selected questionnaires for the study. Key factors include perceived usefulness, perceived ease of use, attitude toward usage and behavioral intention, as shown in Fig. 3.^25^ For carrying out the questionnaires evaluation, 21 revised questionnaires were used to test questionnaire reliability and Cronbach's alpha coefficient with volunteers. Complete data collected from 129 samples also assessed questionnaire sentiment. The researcher administered 21 questionnaires to actual volunteers, assessing questionnaire reliability through Cronbach's alpha coefficient analysis (Table S2).

**Table 2:** Comparison of the previous studies on the technology acceptance model

	Underlying theories	Technology studied	Influential factors	Country	Refs.
1.	Unified models including TAM, UTAUT and PMT	Mobile health service (MHS)	Performance expectancy, effort expectancy, social influence, facilitating condition, threat appraisals	Harbin, China	32
2.	Mixed‐method study with UTAUT and TAM	Telehealth	Intention to use, effort expectancy, perceived privacy, and security, performance expectancy, self‐efficacy	the Netherlands	33
3.	TAM	Smart home technologies	Perceived usability, perceived usefulness, perceived affordability, intention to use, attitudes and willingness to use	South‐western, Virginia, USA	34
4.	Extended UTAUT model	Home telehealth services	Performance expectancy, effort expectancy, facilitating conditions, social influence, doctor's opinion, computer anxiety, perceived security and behavioral intention to use	Slovenia	35
5.	UTAUT2, PMT and PCT	Healthcare wearable device	Perceived expectancy, self‐efficacy, effort expectancy, perceived severity, hedonic motivation, functional congruence, social influence, perceived privacy risk, and perceived vulnerability	Chengdu, China	36
6.	UTAUT	Mobile health (mhealth)	Performance expectancy, effort expectancy, social influence, facilitating condition, users' behavioral intention and use behavior	Dhaka, Bangladesh	37
7.	UTAUT2	Wearable health devices	Health improvement expectancy, effort expectancy, price value, health consciousness, perceived reliability, adoption of wearable health devices	Pakistan	38
8.	SWAM	Smart wearable healthcare devices	Perceived ease of use, perceived usefulness, intention to use, facilitating conditions, compatibility, social influence, perceived social risk and performance risk	Shenzhen, China	39
9.	TAM	Wearable Internet of Things devices	Intrusiveness, comfort, perceived usefulness, perceived ease of use, attitude toward adoption of Internet of Things devices and behavioral intention to use Internet of Things devices.	India	40
10.	TR TRA based TAM	Mobile healthcare technology	Perceived ease of use, perceived usefulness, intention to use, attitude, subjective norms, perceived ubiquity, health knowledge and health need	Taiwan	41
11.	TAM	Long‐term care services model	Performance expectancy, effort expectancy, social influence, facilitating conditions, behavioral intention, use behavior and bandwagon effect	Taiwan	42
12.	VAB and TPB	Mobile health services	Perceived value, attitude, perceived behavior control, technology anxiety and self‐actualization need, behavioral intention and subjective norms	China	43
13.	TPB	Health‐related information and communication technologies	Attitude, perceived behavioral control and subjective norm	USA and Israel	44
14.	TAM motivation model	e‐health application	Health knowledge, information‐seeking and health care need	USA	45
15.	TAM and UTAUT	Long‐term care service system	Perceived ease of use, perceived usefulness, behavioral intention to use and attitude toward using	Khon Kaen, Thailand	Present study

Abbreviations: PCT, privacy calculus theory; PMT, protection motivation theory; SWAM, smart wearable acceptance model; TAM, technology acceptance model; TPB, theory of planned behavior; TRA, theory of released action; UTAUT, unified theory of technology acceptance and use of technology; VAB, value‐attitude‐behavior.

### Statistical analysis

The tool's reliability and statistical hypotheses were assessed through stratified (hierarchical regression) and multiple regression analyses on 129 samples. Tests involved data review, distribution (dispersion) and correlation analysis. Pearson's correlation^29^ was used to determine the relationship between all variables to avoid the occurrence multicollinearity, and the distribution of data was examined by considering the skewness and kurtosis (Figs S3 and S4).^30^ Hypothesis testing was carried out to study technology acceptance and behavior affecting intention to use health data collection and reporting systems with the following hypotheses;

- *Hypothesis 1*. Perceived ease of use positively influences perceived usefulness.
- *Hypothesis 2*. Perceived usefulness positively influences behavioral intention.
- *Hypothesis 3*. Perceived usefulness positively influences attitude toward usage.
- *Hypothesis 4*. Perceived ease of use positively influences attitude toward usage.
- *Hypothesis 5*. Attitude toward usage positively influences behavioral intention in health data collection and instant health reports.

## Results

### Comprehensive analysis of older adults’ health data reports and general participant data in LTC system field visits

This project tested the LTC system to collect health data from 290 older adults in a northeastern municipality, using the LTC application for proactive home screening. The health data of 290 older adult participants were collected, with a median age of 71 years (range 61–96 years; Fig. S5). The LTC system recorded diseases; hypertension and diabetes were prevalent. General analysis encompassed seven variables: sex, age, occupation, education, income, job title and medical center (Table 1). Descriptive statistics and percentages were analyzed, and it was found that among LTC participants, 89.1% were women. The majority of LTC participants (55%) were aged >60 years; only 3.1% were aged 21–30 years. The primary occupation was housewife/butler (44.2%), and education below bachelor's degree (84.5%). Most had an income <15,000 Baht per month (approximately <$420.74 USD) (79.8%) and lived in villages under VHVs' care (79.8%). Nong Yai medical center accounted for most health data (30.2%).

### Analyzing the confidence of the questionnaire used for accepting the LTC system

Coefficients ranged from 0 to 1; values closer to 1 showed high LTC system confidence among older adults (Table S2). By the level that was in the acceptable criteria for various studies, ‘*α*’ must be ≥0.7.^31^ Table S2 shows the questionnaire confidence results from 40 volunteers, yielding an overall confidence of 0.967. Cronbach's coefficients for perceived usefulness, perceived ease of use, attitude toward usage and behavioral intention were 0.928, 0.891, 0.900 and 0.884 respectively. The questionnaire's coefficient (0.884–0.928) meets research criteria. Correlations were established between statements and questions through coefficients. Variables and questions used in technology acceptance assessment were adopted from Table S3. Correlation across all questions (corrected item‐total correlation) ranged from 0.464 to 0.877, similar to Table S4, suggesting retention of all questions for LTC application evaluation.

### Evaluation of questionnaires

The technology acceptance study on the impact of the health data system and instant health report among VHVs took place in northeastern Thailand, involving the statistical analysis of 129 complete questionnaire responses. This analysis included a normality test, where data distribution was examined using skewness and kurtosis, resulting in values within the normal range (between −1.96 and 1.96, as shown in Fig. S3). Furthermore, a Pearson correlation coefficient analysis assessed variable relationships to prevent multicollinearity, showing that all variables had correlation coefficients <0.8, thus minimizing the risk of multicollinearity (Fig. S4).

### Technology acceptance data analysis

The researcher assessed the mean and standard deviation for each question, presenting them with interpretative criteria. Analysis of total scores showed participants' agreement on perceived efficacy, ease of use, attitude and intention, with mean scores of 4.51, 4.29, 4.44 and 4.41 respectively (Table S5). VHVs have undertaken assumption research for hypothesis testing and the findings supported all hypothesis described in section 2.5. Direct relationships were summarized in an influence diagram (Fig. S1); hypotheses were tested (Table S6). Data analysis investigated factors impacting technology acceptance and attitudes influencing VHVs' intention to use the ‘Health Data Collection System’. Linear and multiple regression, using hierarchical regression, were employed. Supplementary details, including Tables S7–S9, offer comprehensive and detailed explanations.

### Comparing studies on technology acceptance and theories among older adults, and analyzing the problems of users in each context

Globally, several technologies support older adult care, including wearables, telehealth, smart homes and assistive technologies. In contrast to previous studies (Table 2), the present study emphasizes introducing a LTC application for Khon Kaen's older adults, evaluating its use. The researcher categorized user‐specific issues and constraints in Table S10. The 23 challenges, grouped into seven major categories, regard health data management issues ubiquitous across health service providers. Addressing these might alleviate broader challenges, such as understaffing, time‐consuming data analysis, heavy workloads, service overload, trust concerns and so on, enhancing overall functioning.

## Discussion

The LTC system was designed to prioritize independence, autonomy, and dignity in LTC for older adults. LTC integrates its application into the existing care system, using smart technology and IoT medical devices. The LTC application consists of four components: mobile app, backend management system, MongoDB data storage system, and a Rest API. This methodology guarantees the effective installation and operation of the LTC application within the existing care system, delivering streamlined data collection, improved healthcare quality, robust data security and scalability. As the key stakeholders, the LTC system relies on various stakeholders, including healthcare professionals, caregivers, elderly individuals, digital health experts, local authorities, data centers, community health volunteers, researchers, regulatory bodies, funders, policymakers and patient advocacy groups. Healthcare professionals collect health data, caregivers access health information and older adults are the primary beneficiaries. Digital health experts design and implement the system, whereas local authorities provide infrastructure and regulatory frameworks. Data centers manage data storage and security, whereas community health volunteers provide data collection and support.

The present study evaluated the adoption of a health data system using questionnaires to assess the factors influencing its use. The LTC application, which allows caregivers and doctors to access medical information through QR code‐enabled smart cards, was evaluated using technology acceptance questionnaires. A sample of 129 individuals, including caregivers and older adult participants, was selected based on age, residency and health status. The data from 290 elderly participants showed the prevalence of diseases, such as hypertension and diabetes, supported by questionnaire confidence and reliability evaluations.

The present study recognized several challenges and limitations to the generalization of the LTC application. These include technical integration challenges, variability in user adoption, data privacy concerns and the complexity of stakeholder engagement. Additionally, there is a recognized need for uniform adoption of the system among healthcare professionals and caregivers, and an understanding of the evolving nature of stakeholder involvement and its implications for long‐term sustainability. These considerations are vital for the successful implementation and efficacy of the LTC system in addressing the needs of the aging population.

The Smart City aims to improve community quality and ecosystems in northeastern Thailand by implementing smart technology models. The LTC system aims to track older adults’ health using intelligent technology and automation. Village volunteers, who are primary carers for older adults, test the system as part of a research project. The study uses TAM to examine the adoption of new technologies and gather information from a sample group of volunteers. The reliability of the tool and the statistical hypothesis were tested using stratified regression analysis and multiple regression analysis. The present study showed that perceived usefulness, ease of use, attitude toward usage and intention to use technology significantly influence participants' perceptions. The LTC system's ease positively impacts usage attitude and intention to use health data, aiming to improve health through fast earth reports and standardized elderly care.

## Disclosure statement

The authors declare no conflict of interest.

## Author contributions

Conceptualization, RP and CC; methodology and validation, RP, CC, WSTT, SM, SC, JL and CP; formal analysis and investigation, RP, CC, WSTT, SM, SC, JL and CP; writing–original draft preparation, CC and WSTT; writing–review and editing, visualization, supervision, project administration, RP and CC. All authors have read and agreed to the published version of the manuscript.

## Ethics statements

This study strictly adheres to ethical standards, approved under HE622163 by Khon Kaen University. Human research follows IRB guidelines (IRB00008614) and Federal Wide Assurance: FWA00003418. It aligns with the Declaration of Helsinki and adheres to the good clinical research practice guidelines (International Council for Harmonization of Technical Requirements for Pharmaceuticals for Human Use Guideline for Good Clinical Practice) as reviewed by the Institutional Review Board.

## Informed consent

Following International Council for Harmonization of Technical Requirements for Pharmaceuticals for Human Use Guideline for Good Clinical Practice, informed consent emphasizes clear understanding of study aspects. Time for questions, voluntary participation, transparency and ethical responsibility uphold participants' autonomy.

## Supporting information


**Figure S1.** Direct correlation influence of various factors According to the research framework diagram.
**Figure S2.** Sample size calculation for the questionnaire study by G*Power package's analysis.
**Figure S3.** Statistical preliminary data by Skewness and Kurtosis.
**Figure S4.** The data analysis of Pearson correlation coefficient.
**Figure S5.** Histogram showing the distribution of the elderly age in Khon Kaen Province.
**Table S1.** The questionnaire was modified from the theory of technology acceptance model. The technology acceptance questionnaire for TAM was as follows: in which 5 means mostly agree, 4 means strongly agree, 3 means moderately agree, 2 means less agree and 1 means not agree.
**Table S2.** Cronbach's Alpha Coefficient of the test questionnaire.
**Table S3.** The variables and questions used in the technology acceptance evaluation of the LTC system.
**Table S4.** The results of the correlation coefficient analysis between the total scores of all questions and questions (Corrected Item‐Total Correlation) (*n* = 40).
**Table S5.** Mean and standard deviation (S.D.) and the level of opinion for each question.
**Table S6.** The results of the hypothesis with test results.
**Table S7.** Linear regression analysis between PU and PE, the perceived usefulness regression analysis statistics of the technology between PU and PE, and linear regression analysis and coefficients between PU and PE.
**Table S8.** Multiple regression analysis of PU and PE variables to AU, the cognitive regression analysis of PU and PE variables to AU, and coefficients for multiple regression analysis.
**Table S9.** Multiple regression analysis of PU and AU, the statistical analysis of the perceived usefulness regression of the technology of PU and AU to BI, and coefficients for multiple regression analysis.
**Table S10.** Summarizes the problems and obstacles in each user context.

## Data Availability

The datasets used and analyzed in this study are available upon reasonable request from the corresponding author.
