# Supplementary material for: Revolutionizing elderly care: Building a healthier aging society through innovative long‐term care systems and assessing the long‐term care acceptance model
Source: Geriatr Gerontol Int. 2024 Apr 7;24(5):477–85. doi: 10.1111/ggi.14856 (PMC11503550; doi:10.1111/ggi.14856)
Supplement: Supplementary file 1 — Figure S1. Direct correlation influence of various factors According to the research framework diagram. Figure S2. Sample size calculation for the questionnaire study by G*Power package's analysis. Figure S3. Statistical preliminary data by Skewness and Kurtosis. Figure S4. The data analysis of Pearson correlation coefficient. Figure S5. Histogram showing the distribution of the elderly age in Khon Kaen Province. Table S1. The questionnaire was modified from the theory of technology acceptance model. The technology acceptance questionnaire for TAM was as follows: in which 5 means mostly agree, 4 means strongly agree, 3 means moderately agree, 2 means less agree and 1 means not agree. Table S2. Cronbach's Alpha Coefficient of the test questionnaire. Table S3. The variables and questions used in the technology acceptance evaluation of the LTC system. Table S4. The results of the correlation coefficient analysis between the total scores of all questions and questions (Corrected Item‐Total Correlation) (n = 40). Table S5. Mean and standard deviation (S.D.) and the level of opinion for each question. Table S6. The results of the hypothesis with test results. Table S7. Linear regression analysis between PU and PE, the perceived usefulness regression analysis statistics of the technology between PU and PE, and linear regression analysis and coefficients between PU and PE. Table S8. Multiple regression analysis of PU and PE variables to AU, the cognitive regression analysis of PU and PE variables to AU, and coefficients for multiple regression analysis. Table S9. Multiple regression analysis of PU and AU, the statistical analysis of the perceived usefulness regression of the technology of PU and AU to BI, and coefficients for multiple regression analysis. Table S10. Summarizes the problems and obstacles in each user context. [file GGI-24-477-s001.docx]

**Supplementary Information**

**Revolutionizing Elderly Care: Building a Healthier Aging Society through Innovative Long-Term Care Systems and Assessing the LTC Acceptance Model**

Chaturapron Chokphukhiao^a,b,c^, Wonn Shweyi Thet Tun^d^, Sakaowrat Masa^c^, Somporn Chaiayuth^e^, Jugsun Loeiyood^f^, Cholatip Pongskul^g^, Rina Patramanon^c*^

^a^ *Information Technology International Program, College of Computing, Khon Kaen University, Thailand, 40002*

^b^*Center of Excellence in Digital Innovation, Faculty of Education, Khon Kaen University, Thailand, 40002*

^c^*Khon Kaen University Phenom Center, Khon Kaen University, Thailand, 40002*

^d^*Department of Chemistry, Faculty of Science, Khon Kaen University, Thailand,40002*

^e^*Division of Public Health and Environment Service, Office of Public Health and Environment, Khon Kaen Municipality, Khon Kaen, Thailand, 40000*

^f^*Division of Information and Communication Technology, Khon Kaen Provincial Health Office, Khon Kaen, Thailand, 40000*

*^g^Department of Medicine, Faculty of Medicine, Khon Kaen University, Thailand, 40002*

* E-mail: narin@kku.ac.th


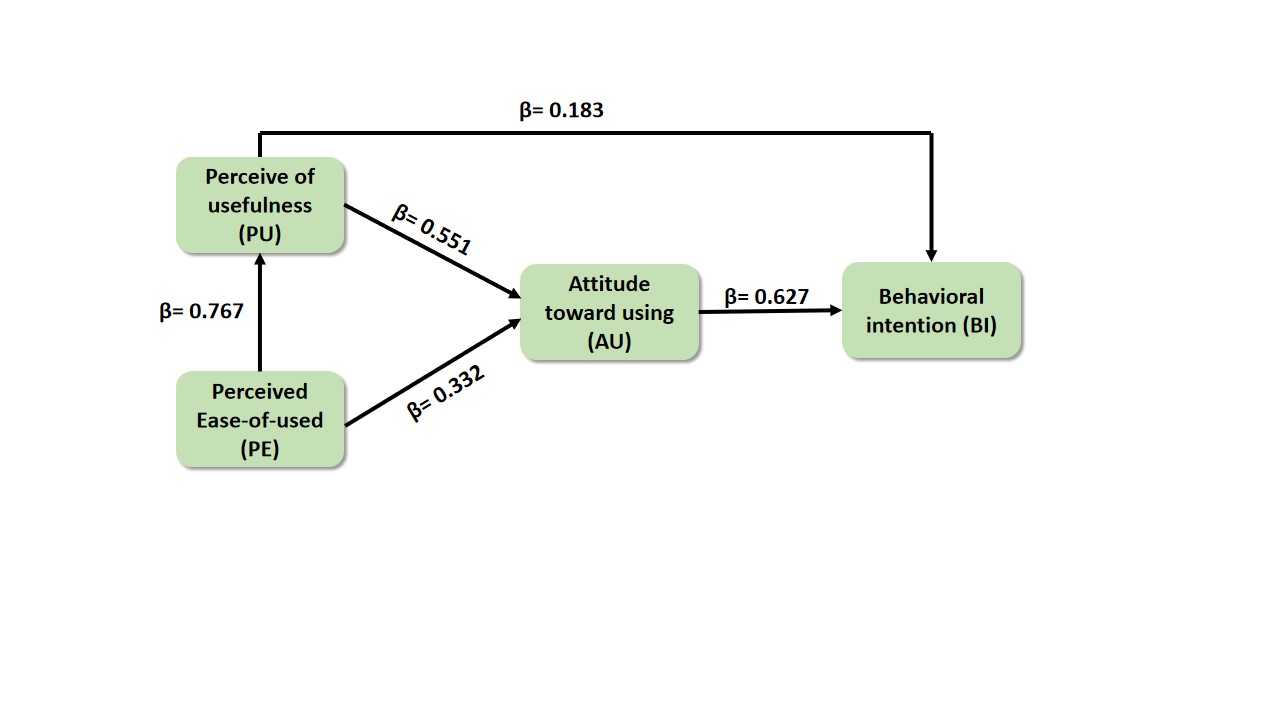


**Figure S1.** Direct correlation influence of various factors according to the research framework diagram.


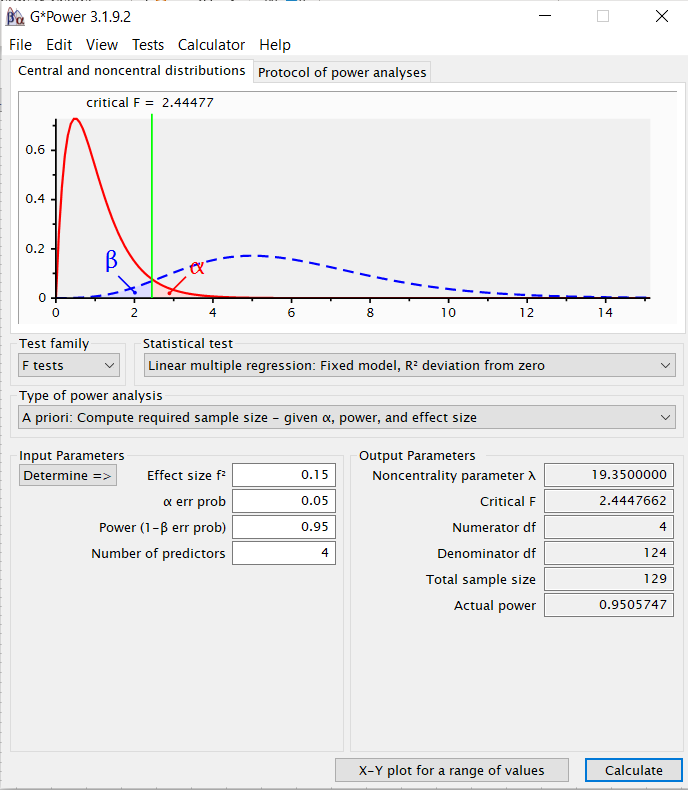


**Figure S2.** Sample size calculation for the questionnaire study by G*Power package's analysis.


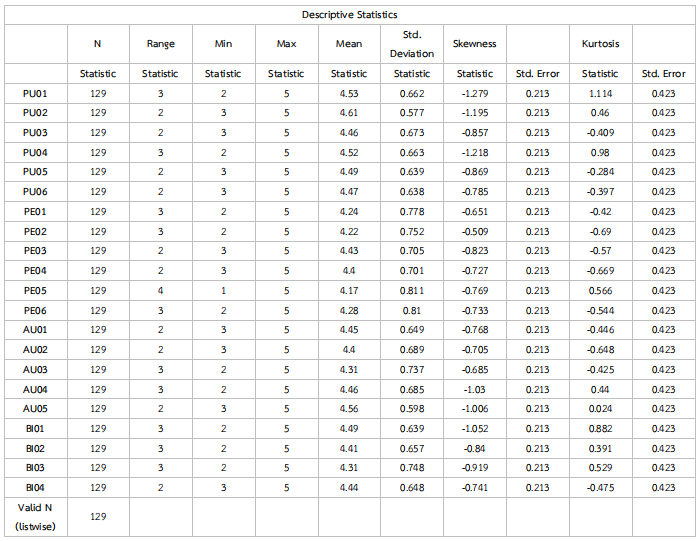


**Figure S3.** Statistical preliminary data by Skewness and Kurtosis.


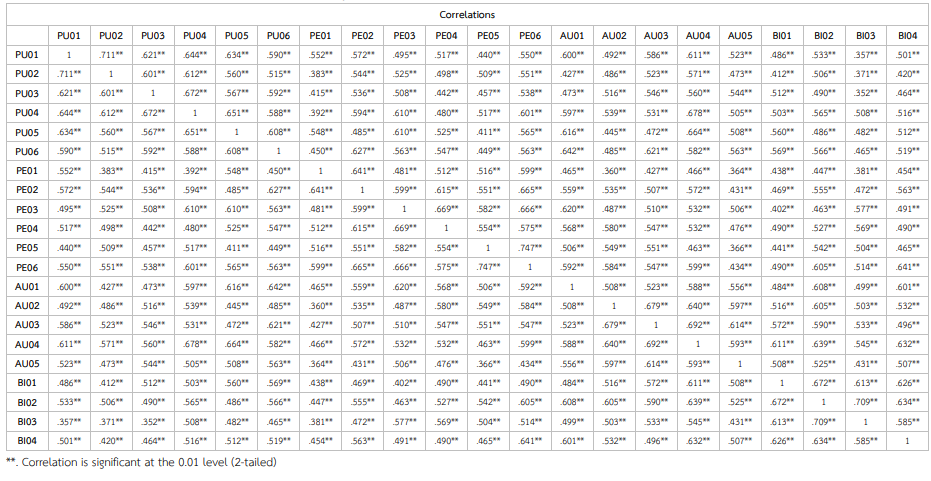


**Figure S4.** The data analysis of Pearson correlation coefficient.


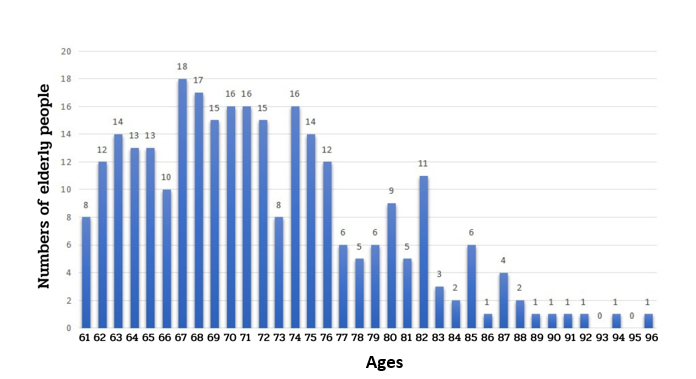
**Figure S5.** Histogram showing the distribution of the elderly age in Khon Kaen Province.

# **Table S1.** The questionnaire was modified from the theory of technology acceptance model^1^. The technology acceptance questionnaire for TAM was as follows: in which 5 means mostly agree, 4 means strongly agree, 3 means moderately agree, 2 means less agree and 1 means not agree.

| **Perceive of usefulness (PU)** |  |  |  |  |  |
| --- | --- | --- | --- | --- | --- |
| 1. Using the LTC system allows you to complete tasks quickly | 5 | 4 | 3 | 2 | 1 |
| 1. LTC system makes your work more efficient | 5 | 4 | 3 | 2 | 1 |
| 1. Using LTC system allows you to work in large quantities | 5 | 4 | 3 | 2 | 1 |
| 1. Using LTC system increases the productivity of your work | 5 | 4 | 3 | 2 | 1 |
| 1. LTC system makes your work easier | 5 | 4 | 3 | 2 | 1 |
| 1. You find LTC system useful for your work | 5 | 4 | 3 | 2 | 1 |
| **Perceive ease of used (PE)** |  |  |  |  |  |
| 1. You can quickly learn how to use LTC system | 5 | 4 | 3 | 2 | 1 |
| 1. You can control LTC system | 5 | 4 | 3 | 2 | 1 |
| 1. The process of collecting health data using LTC system is clear and easy to understand | 5 | 4 | 3 | 2 | 1 |
| 1. The use of LTC system for health data collection is more streamlined than the system that is currently used | 5 | 4 | 3 | 2 | 1 |
| 1. You can easily become an expert in using LTC system | 5 | 4 | 3 | 2 | 1 |
| 1. LTC system is easy to use | 5 | 4 | 3 | 2 | 1 |
| **Attitude towards using (AU)** |  |  |  |  |  |
| 1. Is it good to use LTC system in your work? | 5 | 4 | 3 | 2 | 1 |
| 1. You prefer LTC system to collect data than you currently use | 5 | 4 | 3 | 2 | 1 |
| 1. Using LTC system has positive impact on you | 5 | 4 | 3 | 2 | 1 |
| 1. You think LTC system is valuable for your work | 5 | 4 | 3 | 2 | 1 |
| 1. Do you think the use of LTC system will be popular for the collection of health data information | 5 | 4 | 3 | 2 | 1 |
| **Behavioral Intention (BI)** |  |  |  |  |  |
| 1. Planning to use LTC system in the future | 5 | 4 | 3 | 2 | 1 |
| 1. Try to use LTC system to collect data every time you work | 5 | 4 | 3 | 2 | 1 |
| 1. Plan to use LTC system as a routine to collect data | 5 | 4 | 3 | 2 | 1 |
| 1. You like to use LTC system in your work | 5 | 4 | 3 | 2 | 1 |

# **Table S2.** Cronbach's Alpha Coefficient of the test questionnaire.

| **Factor** | **Cronbach's Alpha Coefficient** | |
| --- | --- | --- |
|  | **n=129** | **Numbers of items** |
| Perceived of usefulness: PU | 0.928 | 6 |
| Perceived Ease of Use: PE | 0.891 | 6 |
| Attitude toward using: AU | 0.900 | 5 |
| Behavioral intention: BI | 0.884 | 4 |
| Total acceptance | 0.967 | 21 |

# **Table S3.** The variables and questions used in the technology acceptance evaluation of the LTC system.

| **Variables** | **Variable names** | **Questions** | **Reference** |
| --- | --- | --- | --- |
| **Perceived of usefulness: PU** | PU 01 | The use of smart technology systems (Long-term care: LTC) helps you get things done quickly. | (F. Davis et al., 1989)^1^ |
|  | PU 02 | Intelligent technology system (Long-term care: LTC) allows your work more efficient. |  |
|  | PU 03 | The use of intelligent technology systems (Long-term care: LTC) allows you to work on large quantities. |  |
|  | PU 04 | The use of intelligent technology systems (Long-term care: LTC) enhances the efficiency of the work. |  |
|  | PU 05 | The smart technology system (Long-term care: LTC) makes your work easier. |  |
|  | PU 06 | The smart technology system (Long-term care: LTC) is useful to your work. |  |
| **Perceived Ease-of-Used (PE)** | PE-01 | Quickly learn how to use smart technology (Long-term care: LTC) | (F. Davis et al., 1989)^1^ |
|  | PE-02 | You can control the intelligent technology system (Long-term care: LTC) to work as you wish. |  |
|  | PE-03 | Do you think that the process of collecting health data using intelligent technology (Long-term care: LTC) has clarity and ease of understanding. |  |
|  | PE-04 | Do you think that health data collection using smart technology (Long-term care: LTC) is more effective? More flexible than traditional health data collection that you are currently using. |  |
|  | PE-05 | Can you be a user? Expertise in the use of intelligent technology (Long-term care: LTC) effectively and easily. |  |
|  | PE-06 | Do you think that smart technology systems (Long-term care: LTC) are easy to use? |  |
| **Attitude towards using (AU)** | AU01 | The use of smart technology (Long-term care: LTC) in your work is good. | (Ajzen & Fishbein, 1972; Lin, Fofanah, & Liang, 2011; Weng, Yang, Ho, & Su, 2018)^2-4^ |
|  | AU02 | You like to use smart technology (Long-term care: LTC) to collect more data than the system you currently in use. |  |
|  | AU03 | The use of smart technology systems (Long-term care: LTC) has a positive influence on you. |  |
|  | AU04 | Do you feel that smart technology (Long-term care: LTC) is valuable for your work? |  |
|  | AU05 | Do you think that the use of intelligent technology (Long-term care: LTC) will be popular to collect automated health data. |  |
| **Behavioral Intention (BI)** | BI 01 | Do you plan to use a smart technology system (Long-term care: LTC) to collect health data in the future? | (Weng et al., 2018)^4^ |
|  | BI 02 | You try to use a smart technology system (Long-term care: LTC) to collect health data every working times. |  |
|  | BI 03 | Do you plan to use a smart technology system (Long-term care: LTC) to efficiently collect health data continuously? |  |
|  | BI 04 | Do you like to use smart technology systems (Long-term care: LTC) in your work? |  |

# **Table S4.** The results of the correlation coefficient analysis between the total scores of all questions and questions (Corrected Item-Total Correlation).

| **Question** | **Corrected Item- Total correlation** |
| --- | --- |
| **Perceived of Usefulness: PU** |  |
| 1. The use of intelligent technology systems (Long-term care: LTC) helps you complete the work quickly. | 0.797 |
| 2. Intelligent technology system (Long-term care: LTC) helps your work more efficient. | 0.821 |
| 3. The use of intelligent technology systems (Long-term care: LTC) allows you to work on large quantities. | 0.701 |
| 4. The use of intelligent technology systems (Long-term care: LTC) enhances the efficiency of the work. | 0.754 |
| 5.The smart technology system (Long-term care: LTC) makes your work easier. | 0.741 |
| 6. The smart technology system (Long-term care: LTC) is useful to your work. | 0.735 |
| **Perceived Ease of Used (PE)** |  |
| 6. The smart technology system (Long-term care: LTC) is useful to your work. | 0.735 |
| 7. Quickly learn how to use smart technology (Long-term care: LTC) | 0.464 |
| 8. You can control the intelligent technology system (Long-term care: LTC) to work as you wish. | 0.741 |
| 9. Do you think that the process of collecting health data using intelligent technology (Long-term care: LTC) has clarity and ease of understanding. | 0.877 |
| 10. Do you think that health data collection using smart technology (Long-term care: LTC) is more effective? More flexible than traditional health data collection that you are currently using. | 0.746 |
| 11. Can you be a user? Expertise in the use of intelligent technology (Long-term care: LTC) effectively and easily. | 0.828 |
| 12. Do you think that smart technology systems (Long-term care: LTC) are easy to use? | 0.806 |
| **Attitude towards Using (AU)** |  |
| 12. Do you think that smart technology systems (Long-term care: LTC) are easy to use? | 0.806 |
| 13. The use of smart technology (Long-term care: LTC) in your work is good. | 0.672 |
| 14. You like to use smart technology (Long-term care: LTC) to collect more data than the system you currently in use. | 0.746 |
| 15. The use of smart technology systems (Long-term care: LTC) has a positive influence on you. | 0.785 |
| 16. Do you feel that smart technology (Long-term care: LTC) is valuable for your work? | 0.823 |
| 17. Do you think that the use of intelligent technology (Long-term care: LTC) will be popular to collect automated health data. | 0.797 |
| **Behavioral Intention (BI)** |  |
| 18. Do you plan to use a smart technology system (Long-term care: LTC) to collect health data in the future? | 0.687 |
| 19. You try to use a smart technology system (Long-term care: LTC) to collect health data every working time. | 0.872 |
| 20. Do you plan to use a smart technology system (Long-term care: LTC) to efficiently collect health data continuously? | 0.824 |
| 21. Do you like to use smart technology systems (Long-term care: LTC) in your work? | 0.788 |

# **Table S5.** Mean and standard deviation (S.D.) and the level of opinion for each question.

| **Question** | **Mean** | **S.D.** | **Comments** |
| --- | --- | --- | --- |
| **Perceived of Usefulness (PU)** |  |  |  |
| PU01: The use of smart technology systems (Long-term care: LTC) helps you get things done quickly. | 4.53 | 0.662 | Accepted |
| PU02: Intelligent technology system (Long-term care: LTC) allows your work more efficient. | 4.61 | 0.577 | Accepted |
| PU03: The use of intelligent technology systems (Long-term care: LTC) allows you to work on large quantities. | 4.46 | 0.673 | Accepted |
| PU04: The use of intelligent technology systems (Long-term care: LTC) enhances the efficiency of the work. | 4.52 | 0.663 | Accepted |
| PU05: The smart technology system (Long-term care: LTC) makes your work easier. | 4.49 | 0.639 | Accepted |
| PU06: The smart technology system (Long-term care: LTC) is useful to your work. | 4.51 | 0.638 | Accepted |
|  | 4.51 | 0.642 | Accepted |
| **Perceived Ease of Use (PE)** |  |  |  |
| PE01: Quickly learn how to use smart technology (Long-term care: LTC) | 4.24 | 0.778 | Accepted |
| PE02: You can control the intelligent technology system (Long-term care: LTC) to work as you wish. | 4.22 | 0.752 | Accepted |
| PE03: Do you think that the process of collecting health data using intelligent technology (Long-term care: LTC) has clarity and ease of understanding? | 4.43 | 0.705 | Accepted |
| PE04: Do you think that health data collection using smart technology (Long-term care: LTC) is more effective? More flexible than the traditional health data collection that you are currently using. | 4.40 | 0.701 | Accepted |
| PE05: Can you be a user? Expertise in the use of intelligent technology (Long-term care: LTC) effectively and easily | 4.17 | 0.811 | Accepted |
| PE06: Do you think that smart technology systems (Long-term care: LTC) are easy to use? | 4.28 | 0.810 | Accepted |
|  | 4.29 | 0.760 | Accepted |
| **Attitude towards Using (AU)** |  |  |  |
| AU01: The use of smart technology (Long-term care: LTC) in your work is good. | 4.45 | 0.649 | Accepted |
| AU02: You like to use smart technology (Long-term care: LTC) to collect more data than the system you currently in use. | 4.40 | 0.689 | Accepted |
| AU03: The use of smart technology systems (Long-term care: LTC) has a positive influence on you. | 4.31 | 0.787 | Accepted |
| AU04: Do you feel that smart technology (Long-term care: LTC) is valuable for your work? | 4.46 | 0.685 | Accepted |
| AU05: Do you think that the use of intelligent technology (Long-term care: LTC) will be popular to collect automated health data. | 4.56 | 0.598 | Accepted |
|  | 4.44 | 0.672 | Accepted |
| **Behavioral Intention (BI)** |  |  |  |
| BI01: Do you plan to use a smart technology system (Long-term care: LTC) to collect health data in the future? | 4.49 | 0.639 | Accepted |
| BI02: You try to use a smart technology system (Long-term care: LTC) to collect health data every working times. | 4.41 | 0.657 | Accepted |
| BI03: Do you plan to use a smart technology system (Long-term care: LTC) to efficiently collect health data continuously? | 4.31 | 0.748 | Accepted |
| BI04: Do you like to use smart technology systems (Long-term care: LTC) in your work? | 4.44 | 0.648 | Accepted |
|  | 4.41 | 0.673 | Accepted |

# **Table S6.** The results of the hypothesis with test results

|  | **Hypothesis** | **Test Results** |
| --- | --- | --- |
| H1 | Recognizing the ease of use of health data collection systems and instant health reports. This positively affects the perceived usefulness (PU) of health data collection systems and real-time health reports | Accept |
| H2 | Perceived Usefulness (PU) of health data collection and instant health reporting systems positively affects the intention to use health data collection and instant health reporting systems. | Accept |
| H3 | Recognizing the Ease of Use (PE) of health data collection systems and instant health reports positively affected Attitudes toward Using (AU) health data collection systems and real-time health reports. | Accept |
| H4 | Attitudes towards Using (AU) health data collection systems and instant health reports positively affect the Behavioral Intention (BI) to use the health data collection system and real-time health reports. | Accept |
| H5 | Attitudes towards Using (AU) health data collection systems and instant health reports have a positive effect on Behavioral Intention (BI) to use the health data collection system and instant health reports | Accept |

The researcher has analyzed the data to study the technology acceptance factors and attitudes that affect intention to use the ‘Health data collection system’ and instant health reports of village volunteers (VHVs) in the area of Khon Kaen municipality by using linear regression analysis and multiple regression analysis using tiered analysis process (Hierarchical Regression), which divides the analysis into 3 parts according to the research framework and meanings of symbols as follows:

**t** is the statistic used to test the hypothesis about the mean of each model in the model.

**B** is the predictor regression coefficient in the equation in raw score form.

**R^2^** is the model efficiency coefficient.

**ß** is the predictor regression coefficient in standard score form.

**Sig.** is a significantly different statistical value.

**Tolerance** is the variance in a variable that cannot be explained by other variables.

**VIF** is the condition of the relationship of the independent variables in the model.

- - - 1. Perceived Ease-of-Used (PE) hypothesis testing affects the perception of usefulness of technology (Perceive of Usefulness: PU)

From Table S7, the researcher performed a regression analysis to determine the direct influence of perceived of usefulness (PU). It was found that the perceived ease of technology had a direct relationship with the perceived usefulness of technology variation equal to 58.9 % (R^2^= 0.589), indicating that the variable perceived ease of use of technology had a positive effect on cognition. Benefits of technology represent 58.9 %, and another 41.1 % is the result of other factors involved. which variables the ease of that technology can be a factor. The perceived usefulness of technology can be determined at the significance level of p = 0.000 as shown in Table S7. When analyzing the regression coefficients of the independent variables in the form of standard scores (ß), it was found that the cognitive variables. Ease of use of technology had a direct influence on the perceived usefulness of technology equal to 0.767 shown in Table S7.

- - - 1. Hypothesis testing of Perceived Ease-of-Used (PE) and Perceived of Usefulness (PU) that affects Attitude toward using (AU)

From Table S8, the researcher performed a regression analysis to determine the direct influence of perceived ease of use of technology. Perceived Ease-of-Used (PE) and Perceived of Usefulness (PU) effects on variables according to the attitude towards using (AU)found that the variables perceived ease of technology and perception. The benefits of technology were directly related to attitude variables to use with a variance of 69.5% (R^2^= 0.695). It shows that the two primary variables affect the dependent variable. Representing 69.5% percent, another 30.5% percent is the result of other factors. The researcher uses a stratified analysis process to find the most suitable model. It was found that the PU and PE variables should be a common variable for both variables in determining Attitude to use is determined from the R^2^ at an increase of 0.045 as Table S8, where both variables can be defined also set the attitude toward use at the significance level of p = 0.000.

In addition, when the regression coefficients of independent variables were analyzed in terms of standard scores (ß). Recognize the ease of use of technology and perceive its usefulness. There was a direct influence on attitude towards usage of technology equal to 0.332 and 0.551, respectively, shown in Table S8, which means that the variables perceived usefulness of technology had a greater positive effect on user attitude than the perceived ease of use of technology. In addition, the researcher also considered the variance inflation factor (VIF) and tolerance to avoid the occurrence of multicollinearity in the case of multiple regression analysis with a high correlation between independent variables, the regression coefficient changes as variables increase Therefore, the researcher considered the relationship between the two independent variables with the following agreement.

1. Tolerance ranges from 0 to 1. If the Tolerance approaches 1, then the source variable is independent and should have greater than 0.4^2^.
2. If the VIF value is 10 or higher, that variable must be excluded from the regression equation. Because the independent variable has the characteristics of a linear relationship with other independent variables^3^.

When considering the Tolerance and VIF in the multiple regression analysis models, they were found to be 0.411 and 2.432, respectively, which shows that the two variables are independent and meet statistical agreement.

- - - 1. Hypothesis testing of Perceive of usefulness (PU) and Attitude toward using (AU) affecting intention to use technology (Behavioral Intention: BI)

From Table S9, the researcher performed a regression analysis to find the direct influence of perceived usefulness of technology (Perceive of usefulness: PU) and attitude towards using (AU) affecting the dependent variables were Behavioral Intention: BI found that the variables perceived benefits of technology and attitude toward use. There is a direct relationship with the intention variables in technology use. A variation of 61.2 % (R^2^ = 0.612) shows that the two source variables affect the dependent variable representing 61.2% percent, and another 38.8% percent is due to other factors involved. The researcher chooses to use a stratified analysis process to find the most appropriate model. It is found that PU and AU variables should be a common variable for both variables in determining set attitude towards use by considering the R^2^-value with an increase of 0.138 where both of these variables can be defined also set the attitude toward use at the significance level p = 0.000 as shown in Table S9 again.

When analyzing the details of each variable, both found that the independent variable perceived benefits of technology as the determinant of the significance level was p = 0.053, and the independent variable attitude towards use was the indicator. at the significance level p = 0.000 (Table S9). In addition, when the regression coefficients of independent variables were analyzed in terms of standard scores (ß), recognizing the usefulness of technology, and the use attitude had a direct influence on intention to use technology equal to 0.183 and 0.627, respectively (Table S9). These results mean that the use attitude variable has a positive influence on the intention to use, the use of more variables perceives the usefulness of technology. Additionally, when considering the Tolerance and VIF in the multiple regression analysis model, it was found that they were equal to 0.351 and 2.852 respectively, indicating that the two variables are independent and meet statistical agreement. These results have been described with the diagram of the direct correlation influence of various factors (figure S1).

# **Table S7.** Linear regression analysis between PU and PE, the perceived usefulness regression analysis statistics of the technology between PU and PE, and linear regression analysis and coefficients between PU and PE.

| Model summary | | | | | | | | | |
| --- | --- | --- | --- | --- | --- | --- | --- | --- | --- |
| Model | R | R Square  (R^2^) | Adjusted R Square | Std. Error of  the Estimate | Change Statistics |  |  |  |  |
|  |  |  |  |  | R Square Change | F Change | df1 | df2 | Sig.F  Change |
| 1 | .767^a^ | 0.589 | 0.586 | 0.34001 | 0.589 | 181.82 | 1 | 127 | 0 |
| a. Predictors: (Constant), MEAN_PE | | | | | | | | | |
| ANOVA^b^ | | | | | | | | | |
| Model |  | Sum of Squares | df | Mean Square | F | Sig. |  | |  |
| 1 | Regression | 21.019 | 1 | 21.019 | 181.82 | .000^a^ |  | |  |
|  | Residual | 14.682 | 127 | 0.116 |  |  |  | |  |
|  | Total | 35.701 | 128 |  |  |  |  | |  |
| a. Predictors: (Constant), MEAN _PE  b. Dependent Variable: MEAN _PU | | | | | | | | | |
| Coefficients^a^ | | | | | | | | | |
| Model |  | Unstandardized  Coefficients | | Standardized  Coefficients | t | Sig. | Collinearity  Statistics | | |
|  |  | B | Std.Error | β |  |  | Tolerance | | VIF |
| 1 | (Constant) | 1.709 | 0.21 |  | 8.316 | 0 |  | |  |
|  | Mean_PE | 0.654 | 0.048 | 0.767 | 13.484 | 0 | 1 | | 1 |
| a. Dependent Variable: MEAN_PU | | | | | | | | | |

# **Table S8.** Multiple regression analysis of PU and PE variables to AU, the cognitive regression analysis of PU and PE variables to AU, and coefficients for multiple regression analysis.

| Model summary | | | | | | | | | |
| --- | --- | --- | --- | --- | --- | --- | --- | --- | --- |
| Model | R | R Square  (R^2^) | Adjusted  R Square | Std. Error of  the Estimate | Change Statistics |  |  |  |  |
|  |  |  |  |  | R Square Change | F Change | df1 | df2 | Sig.F Change |
| 1 | .806^a^ | 0.649 | 0.647 | 0.32972 | 0.649 | 235.14 | 1 | 127 | 0 |
| 2 | .834^b^ | 0.695 | 0.69 | 0.30883 | 0.045 | 18.75 | 1 | 126 | 0 |
| a. Predictors: (Constant), MEAN _PU  b. Predictors: (Constant), MEAN _PU, MEAN _PE | | | | | | | | | |
| ANOVA^b^ | | | | | | | | | |
| Model |  | Sum of Squares | df | Mean Square | F | Sig. |  | | |
| 1 | Regression | 25.563 | 1 | 25.563 | 235.148 | .000^a^ |  |  |  |
|  | Residual | 13.806 | 127 | 0.109 |  |  |  |  |  |
|  | Total | 39.37 | 128 |  |  |  |  |  |  |
| 2 | Regression | 27.352 | 2 | 13.676 | 143.389 | .000^b^ |  |  |  |
|  | Residual | 12.018 | 126 | 0.095 |  |  |  |  |  |
|  | Total | 39.37 | 128 |  |  |  |  |  |  |
| a. Predictors: (Constant), MEAN_PU  b. Predictors: (Constant), MEAN_PU, MEAN_PE  c. Dependent Variable: MEAN_AU | | | | | | | | | |
| Coefficients ^a^ | | | | | | | | | |
| Model |  | Unstandardized Coefficients | | Standardized Coefficients | t | Sig. | Collinearity  Statistics | | |
|  |  | B | Std.Error | β |  |  |  |  | |
|  |  |  |  |  |  |  | Tolerance | VIF | |
| 1 | Constant) | 0.615 | 0.251 |  | 2.454 | 0.15 |  |  | |
|  | Mean_PU | 0.846 | 0.055 | 0.806 | 15.335 | 0 | 1 | 1 | |
| 2 | (Constant) | 0.549 | 0.235 |  | 2.331 | 0.021 |  |  | |
|  | Mean_PU | 0.578 | 0.081 | 0.551 | 7.176 | 0 | 0.411 | 2.432 | |
|  | Mean_PE | 0.297 | 0.069 | 0.332 | 4.331 | 0 | 0.411 | 2.432 | |
| a. Dependent Variable: MEAN_AU | | | | | | | | | |

# **Table S9.** Multiple regression analysis of PU and AU, the statistical analysis of the perceived usefulness regression of the technology of PU and AU to BI, and coefficients for multiple regression analysis.

| Model summary | | | | | | | | | |  |
| --- | --- | --- | --- | --- | --- | --- | --- | --- | --- | --- |
| Model | R | R Square (R^2^) | Adjusted  R Square | Std. Error of  the Estimate | Change Statistics |  |  |  |  |  |
|  |  |  |  |  | R Square Change | F  Change | df1 | df2 | Sig.F  Change |  |
| 1 | .689^a^ | 0.474 | 0.47 | 0.41864 | 0.474 | 114.54 | 1 | 127 | 0 |  |
| 2 | .782^b^ | 0.612 | 0.606 | 0.36095 | 0.138 | 44.838 | 1 | 126 | 0 |  |
| a. Predictors: (Constant), MEAN_PU  b. Predictors: (Constant), MEAN_PU, MEAN_AU | | | | | | | | | |  |
| ANOVA^b^ | | | | | | | | | |  |
| Model |  | Sum of Squares | df | Mean Square | F | Sig. |  | | |  |
| 1 | Regression | 20.074 | 1 | 20.074 | 114.54 | .000^a^ |  | | |  |
|  | Residual | 22.258 | 127 | 0.175 |  |  |  | | |  |
|  | Total | 42.331 | 128 |  |  |  |  | | |  |
| 2 | Regression | 25.916 | 2 | 12.958 | 99.458 | .000^b^ |  | | |  |
|  | Residual | 16.416 | 126 | 0.13 |  |  |  | | |  |
|  | Total | 42.331 | 128 |  |  |  |  | | |  |
| a. Predictors: (Constant), MEAN_PU  b. Predictors: (Constant), MEAN_PU, MEAN_AU  c. Dependent Variable: MEAN_BI | | | | | | | | | |  |
| Coefficients ^a^ | | | | | | | | | |  |
| Model |  | Unstandardized Coefficients | | Standardized Coefficients | t | Sig. | Collinearity  Statistics | | |  |
|  |  |  |  |  |  |  |  |  |  |  |
|  |  | B | Std.Error | β |  |  | Tolerance | VIF | |  |
| 1 | Constant) | 1.029 | 0.318 |  | 3.232 | 0.002 |  |  | |  |
|  | Mean_PU | 0.75 | 0.07 | 0.689 | 10.702 | 0 | 1 | 1 | |  |
| 2 | (Constant) | 0.629 | 0.281 |  | 2.238 | 0.027 |  |  | |  |
|  | Mean_PU | 0.199 | 0.102 | 0.183 | 1.955 | 0.053 | 0.351 | 2.852 | |  |
|  | Mean_PE | 0.65 | 0.097 | 0.627 | 6.696 | 0 | 0.351 | 2.852 | |  |
| a. Dependent Variable: MEAN_BI | | | | | | | | | |  |

# **Table S10.** Summarizes the problems and obstacles in each user context.

|  | **Elderly** | **VHVs/ CG** | **CM** | **Scholars from public health** | **Public health and environment service** | **Public health office** |
| --- | --- | --- | --- | --- | --- | --- |
| 1. Communication problems between patients and their relatives |  |  |  |  |  |  |
| 2. Problems dealing with health information |  |  |  |  |  |  |
| 3. The problem of innate technology resources such as mobile phones, internet, etc. |  |  |  |  |  |  |
| 4.Trust issues between volunteers and service recipients or public health service providers |  |  |  |  |  |  |
| 1. Excessive workload, such as giving consultation with relatives of patients on a case-by-case basis and organize health information performance proactive every day |  |  |  |  |  |  |
| 6. Insufficient medical personnel and taking time to analyze the data |  |  |  |  |  |  |
| 7. The problem of long queues at the hospital |  |  |  |  |  |  |

**References**

(1) Davis, F. D. Perceived Usefulness, Perceived Ease of Use, and User Acceptance of Information Technology. MIS Q. **1989**, 319–340.

(2) Ajzen, I.; Fishbein, M. Attitudes and Normative Beliefs as Factors Influencing Behavioral Intentions. *J. Pers. Soc. Psychol.* **1972**, *21* (1), 1.

(3) Lin, F.; Fofanah, S. S.; Liang, D. Assessing Citizen Adoption of E-Government Initiatives in Gambia: A Validation of the Technology Acceptance Model in Information Systems Success. *Gov. Inf. Q.* **2011**, *28* (2), 271–279.

(4) Weng, F.; Yang, R.-J.; Ho, H.-J.; Su, H.-M. A TAM-Based Study of the Attitude towards Use Intention of Multimedia among School Teachers. *Appl. Syst. Innov.* **2018**, *1*, 36. https://doi.org/10.3390/asi1030036.
